# Ampicillin plus ceftriaxone therapy against Enterococcus faecalis endocarditis: A case report, guidelines considerations, and literature review

**DOI:** 10.1016/j.idcr.2022.e01462

**Published:** 2022-03-02

**Authors:** Andrea Marino, Antonio Munafò, Aldo Zagami, Manuela Ceccarelli, Edoardo Campanella, Federica Cosentino, Vittoria Moscatt, Giuseppina Cantarella, Rosaria Di Mauro, Renato Bernardini, Giuseppe Nunnari, Bruno Cacopardo

**Affiliations:** a Unit of Infectious Diseases, Department of Clinical and Experimental Medicine, ARNAS Garibaldi Nesima Hospital, University of Catania, Catania, Italy; b Unit of Infectious Diseases, Department of Clinical and Experimental Medicine, University of Messina, Messina, Italy; c Department of Biomedical and Biotechnological Science, School of Medicine, University of Catania, 95123 Catania, Italy; d Unit of Clinical Toxicology, Policlinico G. Rodolico, School of Medicine, University of Catania, 95123 Catania, Italy

**Keywords:** Infective endocarditis, Enterococcus faecalis, Double beta-lactams therapy, Ampicillin plus ceftriaxone, Antibiotics resistance

## Abstract

Enterococcus faecalis infective endocarditis (EFIE) continues to be a very serious disease, showing considerable morbidity and mortality rates which are influenced by the spread of multi-drug resistant strains occurred in the last decades. Although aminoglycosides were considered the treatment of choice of EIFE, in recent years several studies have investigated alternative therapeutic approaches, including combinations of beta-lactams, mainly because of the aminoglycoside-renowned nephrotoxicity and the widespread development of high-level aminoglycosides resistance (HLAR). In this scenario, we reported a case involving a prosthetic valve infective endocarditis caused by an aminoglycoside-resistant E. faecalis strain which was successfully treated with ampicillin plus ceftriaxone despite the presence of artificial heart valve and the patient’s severe clinical conditions.

## Introduction

Enterococcus faecalis infective endocarditis (EFIE) is a major clinical problem, accounting for the third most frequent cause of both native and prosthetic valve IE in the community and for the second cause of healthcare-associated infective endocarditis (HAIES) [1], [2]. In the last two decades, this disease has become increasingly prevalent among the elderly and patients with relevant comorbidities, making the treatment of this infection particularly difficult and keeping mortality rates stable [3], [4]. The lack of reliable bactericidal activity of most antimicrobials, the renowned nephrotoxicity arising from the synergistic therapeutic combination of beta-lactams and aminoglycosides, and the widespread development of high-level aminoglycosides resistance (HLAR), pose challenges to obtaining a favorable clinical outcome [5], [6]. This scenario has prompted efforts to identify different pharmaceutical effective solutions for the treatment of EFIE [7].

In vitro and in vivo experimental studies reported synergism of dual beta-lactam combination against clinical strains of E. faecalis, regardless of their susceptibility to aminoglycosides [8], [9]. Specifically, the basis for the synergistic activity of double beta-lactam combination appears to be related to the differential and complementary saturation of E. faecalis Penicillin-binding proteins (PBPs), thus generating the necessary bactericidal effect [10], [11]. Thereafter, clinical evidence supported these experimental results, leading to an update of EFIE treatment guidelines.

Here, we report the case of a patient with prosthetic EFIE treated with dual beta-lactams combination therapy and discuss this therapeutic challenge, along with literature evidence.

## Case description

A 68-year-old woman was brought to the hospital with 7 days of high fever (up to 40 °C) associated with intense asthenia and malaise.

Three months prior, due to a severe and symptomatic aortic stenosis, the patient underwent aortic valve replacement with a biological prosthesis.

Her medical history showed hypertension and smoking. Family history was significant for heart diseases. She took aspirin, ace-inhibitors, beta-blockers, and diuretics.

On admission, she was febrile (T: 38 °C), blood pressure was 130/80 mmHg, heart rate was 90 bpm, oxygen saturation in room air was 97%, respiratory rate was 18 breaths/min.

Physical examination displayed a systolic heart murmur in the aortic auscultation site; her blood test revealed normal white blood test count (6.8 × 10^3^/mm^3^), mild anemia with hemoglobin level of 12 g/dl, normal platelets count (165 × 10^3^/mm^3^). Her inflammatory markers were elevated: CRP was 10 mg/dl (normal range < 0.5 mg/dl), ESR was 80 mm/h (normal range < 10 mm/h). Procalcitonin was negative. Creatinine was 0.7 mg/dl (eGFR with CKD-EPI was 89 ml/min). Normal liver markers (AST, ALT, bilirubin, INR). HIV, HBV, and HCV serology tested negative. SARS-CoV2 test was negative [12], [13].

Three sets of blood cultures and urine cultures were performed and then a Transesophageal Echocardiogram (TTE) followed by a Transesophageal Echocardiogram (TEE) were obtained which showed a small vegetation (< 1 cm) on the prosthetic aortic valve.

Empiric antibiotic therapy was started with ampicillin/sulbactam 3 g three times daily IV, with an infusion time of at least 3 h, plus gentamicin 3 mg/kg/daily in single administration IV as well as daptomycin 10 mg/kg/daily IV.

After two days, fever still persisted and inflammatory markers were higher (CRP 19 mg/dl); procalcitonin was negative. Urine culture was negative. All sets of blood cultures, taken on admission, resulted positive for Enterococcus faecalis resistant to aminoglycosides (resistance pattern as shown in Table 1). Colonoscopy did not show any pathological lesions to justify enterococcal bacteremia. Respiratory pathogen panel test (BIOFIRE® Respiratory 2.1 Panel) tested negative for viral superinfections. Moreover, the patient exhibited high levels of CPK (342 U/L, with a normal range of 40–180 U/L) along with an increased creatinine value (1 mg/dl, eGFR was 58 ml/min).

**Table 1:** Enterococcus faecalis Antibiogram and EUCAST Breakpoints.

Antibiotics	MIC (mg/L)	MIC Breakpoints (mg/L)	
		S	R
AMPICILLIN	4	4	8
AMOXICILLIN	8	4	8
AMOXICILLIN/CLAVULANATE	2	4	8
AMPICILLIN/SULBACTAM	2	4	8
DAPTOMYCIN	2	4	8
VANCOMYCIN	2	4	4
TEICOPLANIN	< 2	2	2
IMIPENEM	4	0,001	4
GENTAMYCIN SCREENING	> 500	< 128	> 128
LINEZOLID	4	4	4
TIGECYCLINE	> 2	0,25	0,25
CIPROFLOXACIN	> 4	4	4
LEVOFLOXACIN	> 4	4	4

Because of the antibiogram and blood tests results, daptomycin was stopped together with gentamicin. We thus initiated ceftriaxone 2 g every 12 h along with ampicillin/sulbactam at the previous dosage.

In the 72 h following the new antibiotic treatment, fever disappeared, inflammatory markers started decreasing (CRP 10.5 mg/dl), and blood cultures taken 48 h apart tested negative. Renal function improved (eGFR up to 80 ml/min) and CPK levels decreased (206 U/L).

We continued ampicillin plus ceftriaxone therapy for 6 weeks after the change in antibiotic regimen, obtaining gradual inflammatory markers decline, clinical improvement and, especially, a new TTE that revealed the dissolution of the vegetation on the prosthetic valve. The patient was then discharged.

Follow-up at 6 months performed with blood tests and TEE showed further amelioration of inflammatory markers and confirmed the dissolution of the vegetation.

## Discussion

Ampicillin plus ceftriaxone (AC) is the combination of choice for HLAR E. faecalis IE and, together with ampicillin plus gentamicin (AG), is one of the two preferred regimes against non-HLAR EIFE [14].

Several studies showed that the typical EIFE patient is elderly, presenting more comorbidities (especially chronic renal failure) than other etiologies IE patient; because of that, non-aminoglycoside-based regimens were tested, mainly due to possible nephrotoxic complications of prolonged aminoglycoside administration [15], [16]. Furthermore, even though aminoglycoside-based regimens have been the first choice for EFIE since the 1950s, the growing prevalence of aminoglycoside-resistant strains has required different therapeutic approaches [17], [18].

Synergistic findings between ampicillin and ceftriaxone against HLAR E. faecalis were reported by Gavalda et al., suggesting that the association with ceftriaxone extends the range of ampicillin’s bactericidal concentrations [9]. These findings confirmed the work of Mainardi et al. about in vitro synergy between amoxicillin and cefotaxime against strains of E. faecalis, showing the decrease in MIC values for amoxicillin in the presence of cefotaxime and vice versa [8], which is explained by the differential targeting of the PBPs by different beta-lactams, with significant synergism against E.faecalis cell wall synthesis [19].

An observational, multicenter, open-label clinical trial conducted by Gavalda et al. showed a clinical cure rate of 67.4% as regards the treatment with ampicillin (2 g every 4 h) plus ceftriaxone (2 g every 12 h) in patients with EIFE, regardless of the presence of HLAR strains, and a treatment-related mortality rate in HLAR EIFE patients of 28.6% (comparable to those reported in previous studies). Furthermore, only 2 patients had treatment-related side effects, but nephrotoxicity was not observed. Despite limitations, this study provided evidence that supports the use of double beta-lactam combination as an effective treatment for patients with HLAR EFIE and, in addition, as a wise option for patients at high risk of developing nephrotoxicity, regardless of strain susceptibility [20].

With the goal of assessing the efficacy and safety of ampicillin plus ceftriaxone combination in the treatment of EIFE compared with ampicillin plus an aminoglycoside, Fernandez-Hidalgo et al. conducted a large, non-randomized, non-blinded, comparative, multicenter cohort study including 87 patients treated with ampicillin plus gentamicin (A + G) and 159 patients treated with ampicillin plus ceftriaxone (A + C). The authors found no differences in mortality, clinical failure, or relapse rates between the two treatment arms during treatment or at 3 months of follow-up, even though A + C-treated patients were in poorer general condition before acquiring the infection than A + G patients. As for adverse events, owing to renal impairment, a higher proportion of A + G patients switched or stopped gentamicin due to renal failure and did not receive the complete course of aminoglycoside-containing regimen. Despite limitations given by retrospective data collection, the lack of a random assignment and the liberal and misleading definition of acute renal failure, with the interruption of aminoglycoside therapy left to the physicians’ choice, this study provided useful clinical data to support the use of A+C regimen for the treatment of EIFE [21].

Pericas et al. conducted a monocenter retrospective analysis of prospective cohort of EFIE patients treated from 1997 to 2011 which showed, similarly to Fernandez-Hidalgo et al., no significant difference in in-hospital mortality (27% vs 23%), 1-year mortality (29% vs 26%), and relapse rate (2 vs 3) between patients treated respectively with A + G and those treated with A + C. Noteworthily, patients who received A+G required a therapeutic switch to A+C due to higher incidence of renal failure during treatment. Furthermore, through the collection and analysis of epidemiological data, this study showed an overwhelming increase of EFIE caused by HLAR strains over the course of the last years, along with the increasing employment of A+C therapy [22].

This evidence provided the rationale for an update in the American Heart Association IE treatment guidelines [23] together with the guidelines of European Society of Cardiology [11] that supports the use of A + C combination as a treatment option for EFIE in patients with HLAR strains and as a tactical alternative in those with impaired renal function and/or at high risk of nephrotoxicity due to aminoglycoside regimen, regardless of HLAR status.

Another retrospective cohort study conducted by El Rafei et al. supported double beta-lactam combination as a safe alternative to A + G for treating EFIE, irrespective of aminoglycoside susceptibility [24].

Moreover, Ramos-Martinez et al. performed a prospective multicenter cohort study to compare the efficacy of a shorter course of A + C (4 weeks) with the recommended duration (6 weeks) for the treatment of native valve EFIE. Notwithstanding the small size of the sample and the lack of randomization, this study provided significant results supporting the shorter treatment course as an alternative regimen due to similar rates of relapse and mortality between treatment groups, especially in patients with a briefer duration of symptoms and those without perivalvular abscess [25].

The clinical data evaluating the usefulness of A+C regime are summarized in Table 2.

**Table 2:** Clinical data evaluating dual beta-lactam combination therapy in EFIE treatment.

Author	Title	Subjects	Method	Regimen	Main finding
Gavaldà et al. [20]	**Brief Communication: Treatment of*****Enterococcus faecalis*****Endocarditis with Ampicillin plus Ceftriaxone**	43 patients with EFIE	Observational, open label, non-randomized, multicenter clinical trial observing outcomes in patients receiving ampicillin plus ceftriaxone treatment	Ampicillin 2 g q4h plus ceftriaxone 2 g q12h	The combination of ampicillin and ceftriaxone is effective and safe for treating HLAR EFIE and could be a reasonable alternative for patients with non-HLAR EFIE who are at increased risk for nephrotoxicity
Fernandez-Hidalgo et al. [21]	**Ampicillin Plus Ceftriaxone Is as Effective as Ampicillin Plus Gentamicin for Treating*****Enterococcus faecalis*****Infective Endocarditis**	246 patients with EFIE	Non-randomized, non-blinded, comparative, multicenter cohort study comparing ampicillin plus ceftriaxone and ampicillin plus gentamicin in patients with endocarditis	Ampicillin 2 g q4h plus ceftriaxone 2 g q12h (n = 159) vs ampicillin 2 g q4h plus gentamicin 3 mg/kg/d (n = 87)	Ampicillin plus ceftriaxone appears as effective as ampicillin plus gentamicin for treating EFIE patients and can be used with virtually no risk of renal failure and regardless of the HLAR status
Pericas et al. [22]	**Changes in the treatment of*****Enterococcus faecalis*****infective endocarditis in Spain in the last 15 years: from ampicillin plus gentamicin to ampicillin plus ceftriaxone**	69 patients with EFIE	Retrospective analysis of prospectively collected data assessing antibiotic resistance, epidemiology and comparing safety and efficacy of ampicillin plus ceftriaxone and ampicillin plus gentamicin in patients with endocarditis	Ampicillin 2 g q4h plus ceftriaxone 2 g q12h (n = 39) vs ampicillin 2 g q4h plus gentamicin 3 mg/kg/d (n = 30)	The prevalence of HLAR EFIE has increased significantly in recent years and that alternative treatment with ampicillin and ceftriaxone is safer than ampicillin plus gentamicin, with similar clinical outcomes.
El Rafei et al. [24]	**Comparison of Dual β-Lactam therapy to penicillin aminoglycoside combination in treatment of*****Enterococcus faecalis*****infective endocarditis**	85 patients with EFIE	Retrospective cohort study comparing safety and efficacy of Dual β-Lactam therapy to penicillin-aminoglycoside combination in patients with endocarditis	Ampicillin 2 g q4h plus ceftriaxone 2 g q12h (n = 18) vs ampicillin 2 g q4h plus gentamicin 3 mg/kg/d (n = 67)	Ampicillin plus ceftriaxone appears to be a safe and efficacious regimen in the treatment of EFIE. Patients treated with this regimen had lower rates of nephrotoxicity and no differences in relapse rate and 1-year mortality as compared to that of the ampicillin plus gentamicin group.
Ramos-Martínez et al. [25]	**Four weeks versus six weeks of ampicillin plus ceftriaxone in*****Enterococcus faecalis*****native valve endocarditis: A prospective cohort study**	109 patients with EFIE	Prospective non-randomized cohort study comparing the efficacy of shorter courses of AC (4 weeks) with respect to the recommended duration of 6 weeks for the treatment of EFIE.	Ampicillin 2 g q4h plus ceftriaxone 2 g q12h for 28 ± 4 days vs ampicillin 2 g q4h plus ceftriaxone 2 g q12h for 42 ± 6 days	Similar rates of relapse and mortality were recorded in patients with native valve EFIE treated with A+C for 4 and 6 weeks, suggesting that a short course of A+C might be sufficient to treat native valve EFIE.

## Conclusion

The case we reported shows the clinical management of a prosthetic valve infective endocarditis caused by a HLAR E. faecalis strain, which was successfully treated with ampicillin plus ceftriaxone, despite the presence of prosthetic valve and the patient’s severe clinical conditions. Furthermore, our patient developed an eGFR reduction probably because of both gentamicin administration and severe infection, as reported in the case series we cited. Although daptomycin represented a valid option to administer as shown on the antibiogram, since the patient had an increase of CPK levels and in order to avoid further renal injuries, we were forced to stop daptomycin infusion and replace it with ceftriaxone. The patient tolerated the dual beta-lactams therapy with no adverse drug reaction, achieving a rapid bacterial clearance on blood cultures and amelioration of inflammation marker levels. Despite the fact that we were forced to switch the antibiotic therapy, our case summarizes the two main indications for the dual beta-lactam combination with ampicillin and ceftriaxone for EFIE, including high risk of impaired kidney function due to aminoglycoside therapy and detection of Enterococcal strain with aminoglycoside resistance (HLAR strains), in spite of rapid microbiological diagnosis, antimicrobial susceptibility testing, and elimination of possible adverse drug reactions. Furthermore, on the basis of susceptibility data, especially on the ampicillin higher MIC than ampicillin/sulbactam or amoxicillin/clavulanate, it is possible to speculate that this E. faecalis was also a penicillinases producer.

This case highlights that it is essential to consider an antibiotic's pharmacodynamic and pharmacokinetic properties in order to choose the most advantageous antibiotics, and not just the antibiogram, to treat challenging infections such as prosthetic valve endocarditis. For instance, the beta lactam time-dependent mechanism of action, which had been optimized by prolonging the infusion time for ampicillin/sulbactam (3 h) and increasing the dosage of ceftriaxone (2 g two times daily), and the synergistic action of both beta lactam antibiotics. Moreover, the epidemiological changes of EFIE with ageing and frail populations, and an underestimation of treatment side-effects, namely the high risk of nephrotoxicity, should force a paradigm shift in the antibiotic choice.

## CRediT authorship contribution statement

**AM** and **AM** wrote the paper. **AZ, EC, FC, VM** and **MC** gave clinical assistance to the case. **RDM** and **GC** searched literature references. **RB, GN** and **BC** revised the paper. All authors read and approved the final manuscript.

## Ethical approval

Not applicable.

## Consent

Written informed consent was obtained from the patient for publication of this case report and accompanying images. A copy of the written consent is available for review by the Editor-in-Chief of this journal on request.

## Conflicts of Interest

The authors declare no conflict of interest.
